# A portable fluorescence spectroscopy imaging system for automated root phenotyping in soil cores in the field

**DOI:** 10.1093/jxb/erv570

**Published:** 2016-01-29

**Authors:** Anton Wasson, Leanne Bischof, Alec Zwart, Michelle Watt

**Affiliations:** ^1^CSIRO Agriculture, Black Mountain Laboratories, Canberra, ACT 2601, Australia; ^2^CSIRO Data61, Canberra, ACT 2601, Australia; ^3^Plant Sciences, Institute of Bio- and Geosciences, Forschungszentrum Juelich, Germany

**Keywords:** Automated, crops, fluorescence, imaging, phenotyping, pre-breeding, productivity, roots, soil, spectroscopy.

## Abstract

Root architecture traits are a target for pre-breeders. Incorporation of root architecture traits into new cultivars requires phenotyping. It is attractive to rapidly and directly phenotype root architecture in the field, avoiding laboratory studies that may not translate to the field. A combination of soil coring with a hydraulic push press and manual core-break counting can directly phenotype root architecture traits of depth and distribution in the field through to grain development, but large teams of people are required and labour costs are high with this method. We developed a portable fluorescence imaging system (BlueBox) to automate root counting in soil cores with image analysis software directly in the field. The lighting system was optimized to produce high-contrast images of roots emerging from soil cores. The correlation of the measurements with the root length density of the soil cores exceeded the correlation achieved by human operator measurements (*R* ^2^=0.68 versus 0.57, respectively). A BlueBox-equipped team processed 4.3 cores/hour/person, compared with 3.7 cores/hour/person for the manual method. The portable, automated in-field root architecture phenotyping system was 16% more labour efficient, 19% more accurate, and 12% cheaper than manual conventional coring, and presents an opportunity to directly phenotype root architecture in the field as part of pre-breeding programs. The platform has wide possibilities to capture more information about root health and other root traits in the field.

## Highlight

Fluorescence imaging was built into a portable box called BlueBox, and roots in soil cores were directly and accurately quantified by automated image analysis, allowing root phenotyping in the field for pre-breeding.

## Introduction

A barrier to breeding for root system architecture has been the lack of high-throughput phenotyping tools for characterizing root system architecture (Trachsel *et al.*, 2010; Wasson *et al.*, 2012), particularly in the field, where characterization is most relevant (Watt *et al.*, 2013). Root architecture characterization of crops in the field has relied on laborious methods such as augur sampling, ingrowth cores, pinboards, and trenching (Oliveira *et al.*, 2000; van Noordwijk *et al.*, 2000). These methods allow only a few genotypes to be compared.

The core-break method was developed to reduce the labour requirements of direct root system sampling, and with it tens of treatments have been compared in the field (Bengough *et al.*, 1992; Bennie *et al.*, 1987; Drew and Saker, 1980; Kücke *et al.*, 1995; van Noordwijk *et al.*, 2000). The core-break method is based upon the relationship between the length of randomly oriented vectors in a volume and the number of intersections (counts) of those vectors with a plane of observation through the volume (Lang and Melhuish, 1970). Plainly, roots are not randomly oriented vectors, and studies have shown that the relationship between intersections and length for roots is altered by the branching and clustering of roots and anisotropic influences (e.g. gravitropism, developmental order of the root, or morphogenetic responses to soil structure) on the direction of growth (Bengough *et al.*, 1992; Grabarnik *et al.*, 1998; van Noordwijk *et al.*, 2000). Thus, the relationship between core-break counts and root length density will vary with the species and soil environment, and must be calibrated empirically for each species and site.

For breeding or pre-breeding efforts, the throughput of direct sampling for root architecture determination must be increased by an order of magnitude above that achieved by the current core-break method. Wasson *et al.* (2014) demonstrated the use of the method for root phenotyping 43 genotypes, but the labour requirements were high and the consistency of the measurements suffered as the number of operators involved increased.

Automation of the core-break methodology would allow a reduction in labour and an improvement in consistency. However, image analysis software would struggle to distinguish roots in an image against a soil background (a problem that also affects human operators). A method must be developed to increase the contrast between roots and soils.

P. H. Gallagher, in an early paper on the fluorescence of soil under UV light (Gallagher, 1949), reported that in 1937 H. L. Richardson had ‘suggested that screened ultra-violet light might prove serviceable in studies of root distribution’, on the basis that roots fluoresced brightly. A study of the roots of 135 species from 65 families of vascular plants found that all but six fluoresced under UV (365nm) light, predominantly emitting a blue colour; species whose roots fluoresced included wheat, barley, oats, and maize (Goodwin and Kavanagh, 1948). The fluorescence is attributed to phenolic compounds (Harris and Hartley, 1980; Hartley and Harris, 1981; Ibrahim and Towers, 1960), including coumarins (Goodwin and Kavanagh, 1949) and flavonoids (Buer *et al.*, 2010; Wasson *et al.*, 2006).

Various studies have explored the links between fluorescence and root traits. The exudation of a fluorescent compound, scopoletin, by oat roots has been used in an assessment of its allelopathic potential (Fay and Duke, 1977). The intensity of root fluorescence was linked to root elongation rate and nutrient uptake in soybean (Dyer and Brown, 1983). UV lights have been included in some minirhizotron systems, with root fluorescence investigated as a marker of root age and function, with mixed results (Smit and Zuin, 1996; Wang *et al.*, 1995). Root fluorescence has been shown to be altered by microbial colonization (Gamalero *et al.*, 2004; Holland and Fulcher, 1971). A laser-induced fluorescence imaging system has recently been described for the purposes of investigating root and rhizosphere interactions in rhizoboxes and on filter paper (Alony and Linker, 2013).

In the present study, a fluorescence imaging system for capturing high-contrast core-break images was developed, called BlueBox. The development of the system, described in this article, allows automated in-field phenotyping of root systems.

## Materials and methods

### Plant growth and root sampling

Wheat (*Triticum aestivum* L. cv. Gasgoyin) experiments were grown at the CSIRO Ginninderra Experiment Station in Canberra, Australia (35° 12′28 73″ S, 149° 5′2 03″ E) between May and December 2013. Bulk density varied from 1.3g/cm^3^ at the surface to 1.5–1.6g/cm^3^ between the depths of 80 and 160cm. The experiments were sown on a black alluvial clay soil, which was heavy and sticky when the moisture profile was full, and which cracked as the season progressed. The experiments were managed with prophylactic fungicide and herbicide treatments to prevent weeds and diseases such as rust interfering with crop growth and yield. These experiments were sampled opportunistically for the purposes of this study.

**Fig. 1.**
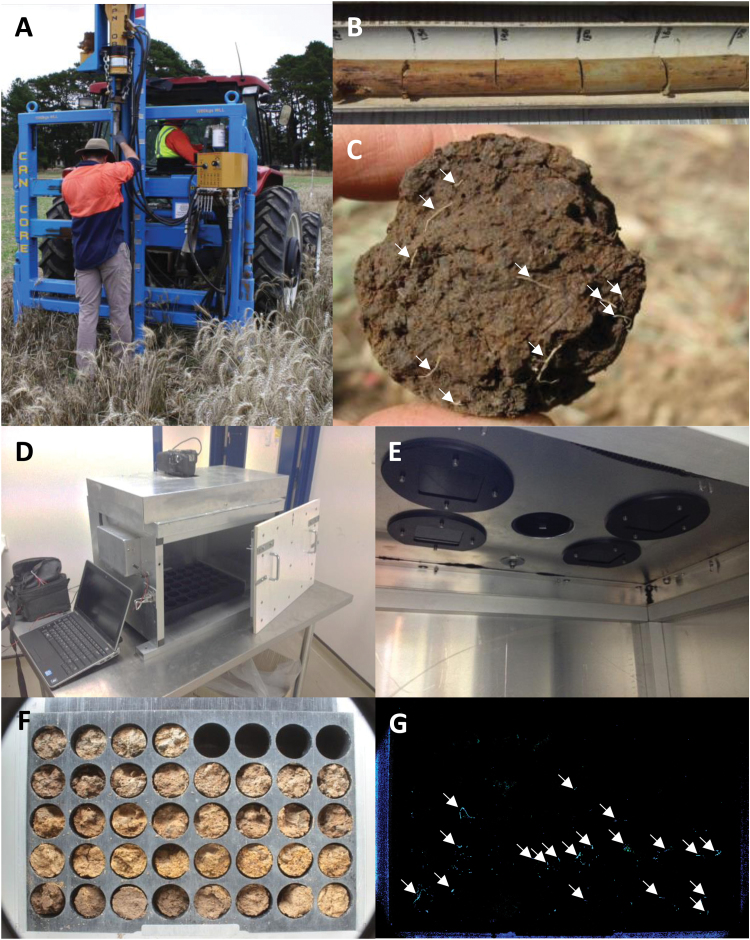
**Steps in the BlueBox methodology.** (A) An operator root sampling with a 2 m long stainless steel coring tube with a tapered tip (not visible). The tube was driven into the ground by a tractor-mounted push press. (B) A soil core emptied into a cradle in a manual root-counting method. The core has been scored with a knife every 10cm to facilitate breaking. (C) The broken face of a soil core segment as seen by a human operator. The number of visible roots (highlighted with white arrows) is assessed in a few seconds. (D) The fluorescence imaging box (in the laboratory; for field use this would typically be mounted on a utility vehicle). The access panel at the front is ajar and a cassette is visible within. The digital SLR camera is operated remotely from the laptop. The battery on the left powers the LEDs, installed underneath the top cover. (E) View of the ceiling of the interior of the box. The central aperture is face of the digital SLR camera lens. The other apertures are UV-emitting LEDs (inactive in this photo) behind black light filter glass. (F) A visible image of the cassette containing the soil core segments. The cassettes were flipped so that both faces of the segment were photographed (the underside is visible here). (G) The fluorescence image of the cassette shown in F. The roots are fluorescing blue; larger roots are highlighted with white arrows.

Soil sampling was performed as described in Wasson *et al.* (2014). In brief, soil cores were collected from within wheat plots using 2 m long, 45mm diameter stainless steel soil-coring tubes driven into the ground by a tractor-mounted hydraulic push press (Fig. 1A). Samples were taken after harvest, within the row and over the residual crown of the harvested plants. The cores were then emptied into cradles and subdivided by depth according to the methods described below. It is important to note that the cores were ‘broken’ into segments and not cut. Breaking the core creates a fracture plane, and the roots passing through that plane were pulled with one of the two opposing faces of the resulting segments, leaving lengths of roots exposed on the faces that were easy to count. Were the segments to be cut, the cut would slice through the roots, leaving only a very small cross-section of each root on each opposing face. These cross-sections would be almost impossible to count or image.

Two experiments were performed: proof-of-concept fluorescence spectroscopy, and calibration and testing of the BlueBox. Soil sampling for the proof-of-concept fluorescence spectroscopy occurred when the crop was booting in late winter; for BlueBox calibration and testing, the sampling occurred at maturity, after the crop had been harvested in summer.

### Proof-of-concept fluorescence spectroscopy of roots and soil

A proof-of-concept experiment, using a fluorescence spectrometer, was performed to confirm that the roots would fluoresce under 365nm UV excitation, and the soil in which they grew would not. This would suggest that a high-contrast image could be obtained using fluorescence techniques.

Soil cores were collected from wheat plots and subdivided into 10cm fragments by depth (Fig. 1B). Subsamples of soil were collected and the roots were then washed from each sample with a hydropneumatic root elutriation system (Smucker, 1982; Wasson *et al.*, 2014). Small subsamples of root and soil were loaded into a UV-transmissive 96-well plate and the fluorescence emissivity between 380 and 710nm was measured on a SpectraMax M2 microplate reader (Molecular Devices, California) at an excitation frequency of 365nm.

### In-field imaging system

A portable fluorescence imaging system (subsequently called the ‘BlueBox’) was designed for use in the field. The system was designed to fit on a utility vehicle. It was fabricated in our site workshop from aluminium sheeting (Fig. 1D) and a light-emitting diode (LED) lighting system was installed inside (Fig. 1E). A Canon EOS 600D digital single-lens reflex (SLR) camera, with a Canon EF-S 18–55mm f/3.5–5.6 III lens (minimum focusing distance of 250mm) was mounted inside the box to capture the images. With an 18 megapixel sensor the images generated would be 5184×3456 pixels in area; approximately 13 pixels per mm of length or 169 pixels/mm^2^. Images were stored in high-quality JPEG format. The size varied with the content of the images, ranging between 1.5 and 9.5 MB.

A 40-well cassette was fabricated from Delrin®, a low-friction plastic that appears to be non-fluorescent at 365nm excitation. The cassette takes 5cm fragments of the soil cores with the broken faces exposed to the UV light and the camera. The cassette was designed with removable lids on the top and bottom so that the cassette could be ‘flipped’ and the underside imaged, capturing both broken faces of the 40 5cm core fragments. The fragments were ordered in the cassettes by depth. The cassettes were designed to be emptied between cores and reused immediately in the field.

Two lighting systems were trialled, with excitation at 447.5nm and 365nm. The 447.5nm excitation is in the visible wavelengths (royal blue) with the emission at lower-energy visible wavelengths (yellow-red), requiring the camera to have a 500nm longpass filter (i.e. a filter that passes wavelengths larger than a particular frequency, and blocks or attenuates those shorter than a particular frequency).

Four SR-01-R0800 Royal-Blue Rebel LEDs mounted on 20mm Star CoolBase Metal Core Printed Circuit Boards (MCPCBs; 890 mW at 700 mA; Luxeon Star LEDs, Ontario, Canada) were used to achieve a 447.5nm excitation maximum. Appropriate dispersion of the light was achieved with a 39 ° 20mm Optic Lens (Fraen Corporation, USA). The light was filtered with #4600 Medium Red Blue Rosco dichroic permacolour filters (Rosco, Stamford, Connecticut) to prevent leakage into the emission wavelengths. These filters have a transmission at 480nm of 11% and at 500nm of 2%. The fluorescence emission at these wavelengths occurs at wavelengths longer than 500nm. The camera was fitted with a Y-50 (500nm) Long Pass Filter using a 2-inch Square Threaded Filter Holder for Imaging Lenses and a M52 to M58 Filter Thread Adapter (Stock no. NT59-445, NT59-447, NT66-061; Edmund Optics, Singapore).

Excitation at 365nm (UV) was not visible. Four LZ4-40U600 UV LEDs (LED Engin, California) mounted on Standard Star 1 MCPCBs were used to achieve a 365nm excitation maximum. Emission of roots was shown by fluorescence spectroscopy to be centred at 460nm. The UV LEDs were filtered U-360 2-inch square bandpass filters (i.e. filters that allow light within a particular range of frequencies to pass through; Edmund Optics, Singapore) to prevent leakage into the visible wavelengths. Imaging was trialled with and without a 452nm bandpass filter (Edmund Optics, Singapore) for the camera.

### In-field root measurements by coring team

The automated BlueBox was included in the soil coring operations of a team of three to four people, with two operating the push press, and either one or two people breaking the cores into 5cm increments, loading the cassettes and imaging them. The cassettes would then be flipped and imaged again. Both a visible image (with the hatch removed for lighting) and a fluorescence image were obtained for each face of the cassette (Fig. 1F, G). The exposure time was 0.8 seconds for visible images and 6 seconds for fluorescence images. The camera was set to a time variable setting with manual focusing. This meant that the aperture was being set automatically by the camera, resulting in variation in the setting between image types (see Discussion). Cassettes were emptied at the site as soon as the images had been acquired.

### Image analysis software to quantify roots on core faces

**Fig. 2.**
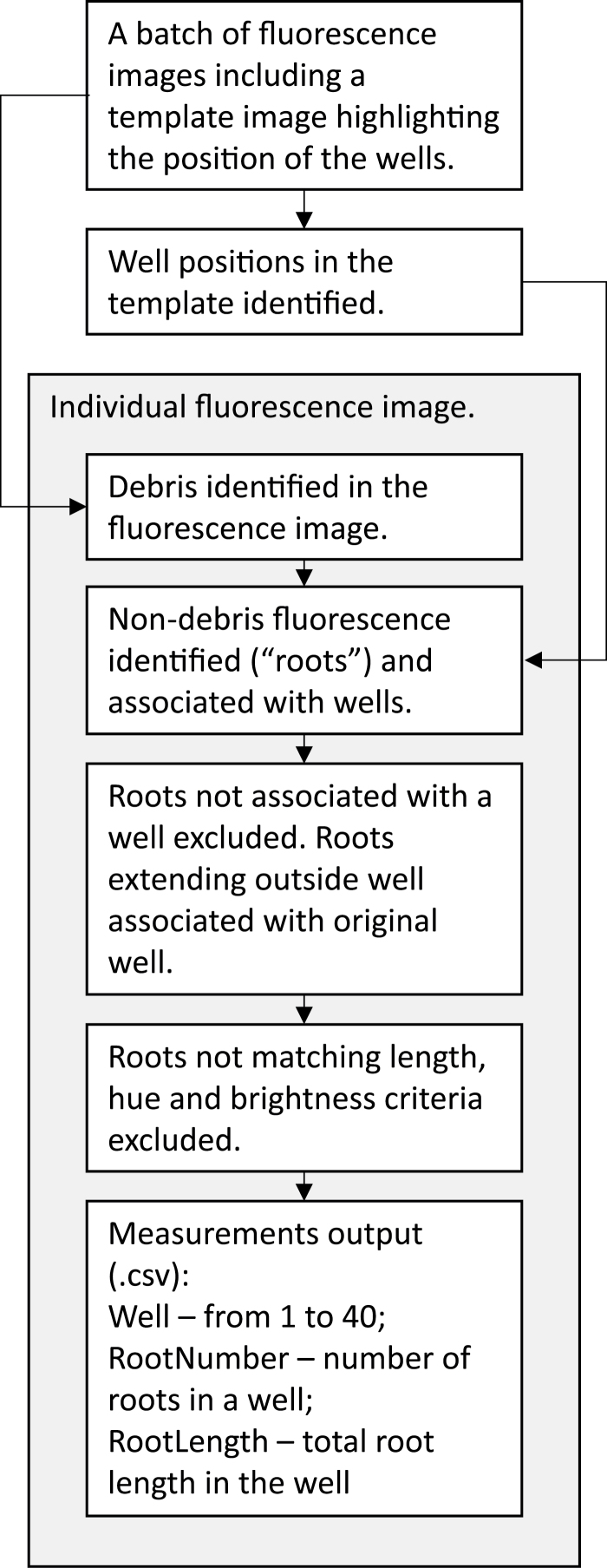
Simplified flowchart of the processing steps undertaken by the image analysis software.

The analysis of BlueBox images of the cores consisted of two steps: (i) finding the wells in the cassette that hold the cores and (ii) measuring the roots within each well. These steps are summarized in Fig. 2.

#### Identification of well positions in the template image

**Fig. 3.**
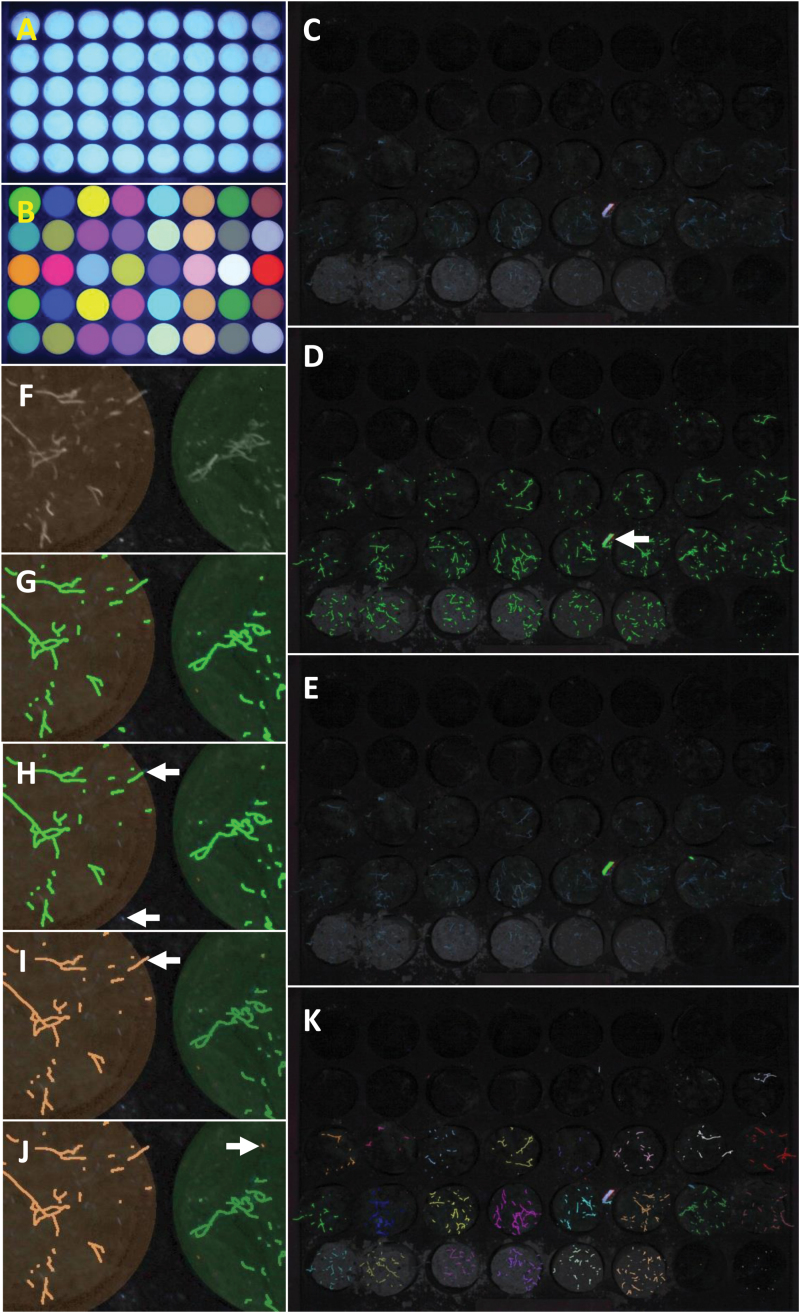
**The image processing steps in the analysis software.** (A) Image of cassette template. (B) Circular ‘Well’ labels (each label is overlaid as a separate colour). (C) RGB image of cores in wells. (D) Detected ‘Linear structures’ mask (overlaid in green on blue channel). (E) ‘Debris’ mask (overlaid in green on original image). (F–J) All subsets shown with ‘Well’ labels as transparent overlays: (F) blue channel image; (G) ‘Debris-excluded root’ mask (in green); (H) ‘Roots within wells’ mask (in green), with arrows indicating excluded roots; (I) labelled ‘Reconstructed roots’ (in ‘Well’ label colour), with arrow indicating a reconstructed root; (J) labelled ‘Filtered roots’ (in ‘Well’ label colour), with arrow indicating a root filtered by hue. (K) Labelled ‘Filtered roots’ (overlaid in ‘Well’ label colour on the RGB image).

Because the cores in the cassette can have variable contrast with the material of the cassette itself, segmentation (the identification of objects in digital images that can be analysed; see Yoo, 2004, for general discussion) of the cores within the image could be problematic. It was more reliable to segment the wells instead. To make this step more straightforward and robust, a template was constructed to match the dimensions of the cassette exactly and to have high contrast between the body of the template and the wells. At the beginning of each day’s work, an image of the template (Fig. 3A) was captured. Because the template is positioned within the BlueBox in the same location as the cassette, there is excellent correspondence between well locations in the template and well locations in the cassette.

The template image was downsampled by the same factor as the roots (described below). Of the red, green, and blue channels, the blue channel gave the greatest contrast between the wells and the body of the template, so segmentation was performed on this channel image.

This downsampled blue channel image was median filtered to reduce noise, prior to filtering to highlight the wells by suppressing any background variation surrounding them. This background suppression was performed using connected component (or attribute) morphological operators (Salembier and Wilkinson, 2009). These operators were used in preference to older structuring element-based morphological functions because they were contour preserving and there is more selectivity in the way the image is modified. In this case, the attributes are that elongation must be less than 1.2 (because the wells are circular), and well area must lie in a specified range.

Because the background-suppressed image had good contrast between the wells and the template, there was no need for sophisticated segmentation techniques. The wells were separated from the background through simple global thresholding. This threshold is determined using an automated thresholding method, based on a bivariate histogram of input grey level versus gradient, as it is more reliable than methods based on the simple image histogram and is used in image normalization (Sintorn *et al.*, 2010).

The ‘Thresholded wells’ regions could be non-circular due to some flare at the edges of the template—an optical artefact (barrel distortion, similar to that utilized by fisheye lenses in consumer photography) caused by the proximity of the camera lens to the subject (short focal distance). So, for each well region, the geometric centre was found and the major axis of the best-fitting ellipse calculated. A circle with this radius and centre at the same point was then constructed as a model for the well extent. The well regions were then labelled in raster order (top left to bottom right in the grid of x and y coordinates in an image) from 1 to 40 (Fig. 3B). As could be seen in a transparent overlay of the resulting well labels (with each label shown as a separate colour), there was a good correspondence between the circular ‘Well’ labels and the actual extent of the wells.

#### Processing individual fluorescence images

Because the root fluorescence signal was not very bright despite the use of long exposure times, background noise (generated in the imaging sensor of the camera) can be significant. Since the spatial resolution was more than adequate to resolve very fine roots, the images were downsampled by a factor of 2 in both x and y directions, averaging over neighbouring pixels and increasing the signal-to-noise ratio (Fig. 3C). The greatest contrast between the roots and the background was in the blue channel, so root detection was performed on this image.

The roots (and any other linear structures) were detected using a fast linear detection algorithm, which is an established technique for neurite outgrowth detection (Vallotton *et al.*, 2007) in several commercial high-throughput cell screening systems. This algorithm detects the roots by finding the ridgelines of high local pixel intensity in the image using directional non-maximum suppression (Sun and Vallotton, 2009). Thus, the segmentation of the roots tended to follow the path a human operator would trace, encompassing the brightest point within broad linear structures. This contrasts with other methods, which use thresholding followed by morphological thinning, or skeletonization. The binary mask of the root-like ‘Linear structures’ is shown in Fig. 3D.

#### Debris identification in the fluorescence image

Debris, such as fragments of straw from the crop residue or lint from the operators’ clothing, frequently contaminated the cassette and fluoresced, potentially becoming confused with the roots where the debris was linear. This was the case with the thick strip of debris indicated by the white arrow in Fig. 3D. The distinguishing feature of the debris is that it was thicker than the roots, and it was on that basis that the debris was segmented. First, the thin bright objects (roots) were removed from the image, leaving the thicker bright debris and background unchanged. The image analysis procedure used was a morphological opening with a polygonal structuring element with a radius of the maximum root radius (Soille, 2003); that is, if a circle-like polygon with the radius of the largest root could fit within a brighter object, then those parts in which the polygon fitted would be retained, and if not, they would be removed (morphological opening). The consequence is that only bright objects that are thicker than roots are considered as part of the foreground in the ‘morphologically opened’ image. Then, the background of this image (i.e. everything except the thick debris) was found by suppressing all linear structures that might be debris. The morphological operation used to produce this ‘background image’ was connected component filtering of objects with the attributes of elongation less than five times the length over the width or area greater than 0.25 × the well area. The ‘background removed’ image was the difference between the ‘morphologically opened’ image and the ‘background’ image. The debris was detected by simple global thresholding of the ‘background removed’ image with a specified threshold of 50. The ‘Debris’ mask is shown in Fig. 3E. A simple logical operation can exclude the lines within the ‘Debris’ mask from the ‘Linear structures’ mask, leaving the ‘Debris-excluded roots’ mask.

#### Association of non-debris fluorescence (‘roots’) with wells and exclusion of debris fluorescence

The aim was to measure, for each core, the numbers and lengths of roots that lie within the well and to include the root portions that originate within the well but extend beyond the well boundaries. Isolated root segments disconnected from the wells were ignored. To illustrate how this was done, consider a subset of the RGB image (Fig. 3F–J). The ‘Well’ labels derived from the template image (Fig. 3B) are shown as a transparent overlay on the subset images in Fig. 3F-J). The first step was to apply the ‘Well’ mask to exclude all roots lying outside this mask. For the blue channel (Fig. 3F), the ‘Debris-excluded roots’ mask (Fig. 3G) was masked by the ‘Wells’ mask to produce a ‘Roots within wells’ mask (Fig. 3H). This excludes both isolated roots lying between wells (lower white arrow) and roots that start within the well and extend beyond the well region (upper white arrow). (Note that the ‘breaking’ of the core into segments leaves roots that extend out of the opposing faces of the segments; these roots can extend outside the well when segments are placed in the cassette.) The portion of the root extending beyond the well (removed by the previous step) can then be reconstructed (white arrow in Fig. 3I) using morphological reconstruction, a standard technique in mathematical morphology. All ‘Reconstructed roots’ were then labelled according to the ‘Well’ to which they belong (Fig. 3I).

#### Exclusion of roots not matching length, hue, and brightness criteria

Finally, the ‘Reconstructed roots’ could be filtered on the basis of their length (must be longer than a specified minimum length), hue (must be blue) and lightness (must be brighter than a specified minimum intensity). For example, the small red root-like structure visible in Fig. 3J has been excluded of the basis of hue (it is red rather than blue).

#### Measurement output

The resulting labelled ‘Filtered roots’ for the full image are shown in Fig. 3K. The ‘Filtered roots’ were measured and the results saved to a file in Comma Separated Variable (CSV) format. The measured fields were:

• Dim – 1 if the image was Dim, 0 otherwise (this parameter is a consequence of the automatic aperture selection by the camera, and would not otherwise be required)
• Core – the well (numbered in raster order from 1–40)
• RootNumber – the number of roots for the well
• RootLength – the total root length for the well (in pixels).

### Calibration of image analysis software: correlation of BlueBox output with root length density

In 2013, 10 (2 m long) soil cores from wheat plots were broken into 5cm segments and imaged on both faces with the BlueBox. The 5cm segments were collected and combined into 10cm depth increment samples. These samples were washed and the root systems isolated (as for fluorescence spectroscopy, described above). These root systems were scanned and the lengths measured to generate root length densities (cm cm^−3^), against which the measurements of the BlueBox were compared.

Scripts were written in the statistical programming language R to link the fluorescence-based root measurements from the BlueBox to the sample information and hence the washed root length densities of the subset of measurements. The combinations of core-break counts (at 5cm increments) within the core, which could be matched with the root length density at any given 10cm depth increment, were also varied. The nature of the fluorescence images varied with the exposure and aperture settings of the camera. The optimum combination of outputs, camera and algorithm settings, and core-break sampling frequencies was empirically determined against the root length density. This included sampling the number of fluorescent detections in empty wells as a measure of false-positive ‘noise’. Root counts for each 5cm increment matching a 10cm washed root sample were found to correlate best with the washed root length density; for example, the sum of the root counts at 20, 25 and 30cm correlates with the washed root length density of the segment from 20–30cm, the sum of the root counts at 30, 35 and 40cm correlates with the washed root length density of the segment from 30–40cm, etc.

#### Comparison with manual methods

The visible images of the soil cores were examined and the number of roots visible in each well were counted manually (hereafter termed the ‘manual core-break’ method). The data from the manual core-break method, analogous to what would occur in the field without the BlueBox, were used for an alternative calibration against the washed root length densities. This allowed the BlueBox calibration to be compared with a manual core-break calibration.

## Results

### Fluorescence spectroscopy suggests that root autofluorescence provides a strong contrast between roots and soil

**Fig. 4.**
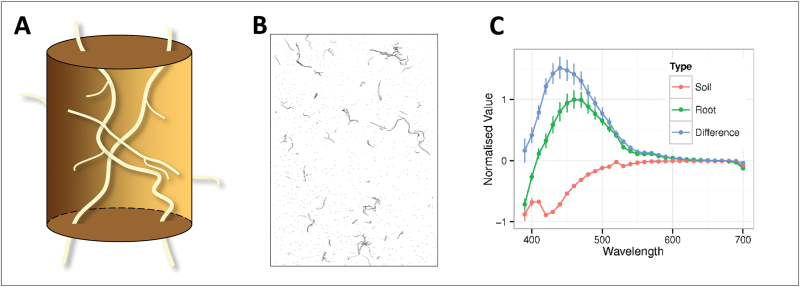
**Fluorescence spectroscopy of roots and soil.** (A) A diagram of roots inside a 5.0cm long × 4.2cm diameter soil core fragment. (B) A subset of roots washed from a 10cm long × 4.2cm diameter soil core fragment from a depth of 20–30cm. The roots have been separated from soil in a hydropneumatic root elutriation system (Smucker, 1982) and imaged on a flatbed scanner in a tray of water. (C) The fluorescence emissivity properties (at 380–710nm) of roots (green) and soil (red) obtained from the experimental site and measured with a fluorescence spectrometer. The values have been normalized against the largest measurement. The positive values represent fluorescence emission and the negative values absorption. The blue line is the difference between the roots and soil at each wavelength. Error bars are standard error of the normalized mean across five replicates. Error bars for the difference were calculated by addition (Taylor, 1997).

Fluorescence spectroscopy of roots and soil from the field was performed to assess the contrast of the two materials. The nature of the samples of roots embedded in soil core segments is shown diagrammatically in Fig. 4A. Roots separated from these samples by root washing are shown in Fig. 4B. The recovered roots are fragmented, probably because only parts of the root system are within the volume extracted in the core, and because some lengths of root will fragment in the root washing process. When excited at 365nm (Fig. 4C), the soil was not fluorescent, and it was absorptive in the shorter wavelengths measured (380–710nm). The roots fluoresced strongly at 460nm, and the peak intensity difference between roots and soil was at 440nm.

### Excitation of the core-break face with UV light produces high-contrast images of roots in soil

**Fig. 5.**
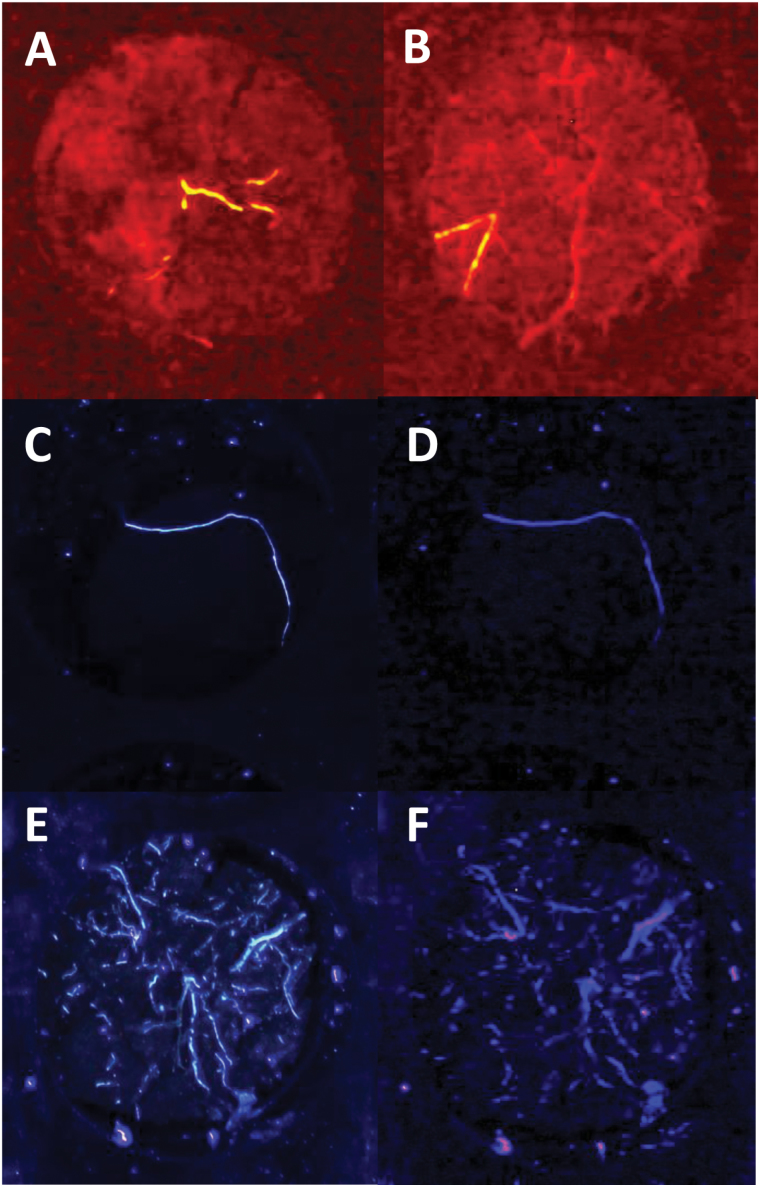
**Development of the imaging system.** (A, B) Exposed core faces illuminated at 445nm and imaged with a 500nm longpass filter. There appears to be reflection from soil components in the image, appearing as a red ‘cloudy’ background. Some roots are seen to fluoresce strongly in a yellow colour. However, there are also linear red structures, which appear largely to be weakly fluorescing roots, but which may also result from features in the soil. (C–F) A comparison of exposed core faces illuminated at 365nm and imaged without (C, E) and with (D, F) a bandpass filter. There is little background reflectance or fluorescence in the images, but the bandpass filter reduces the contrast of the weakly fluorescent roots.

Soil cores were sampled from the field and subdivided into 5cm segments in the BlueBox cassette by depth. An image of a soil core with roots was obtained with 447.5nm excitation and 500nm longpass emission filtering (shown in Fig. 5A, B). Strongly fluorescing roots appeared yellow in colour, in contrast to the background, which was red. Weakly fluorescent roots were visible as segmented linear structures in red and were hard to distinguish from linear features in the soil. The soil appeared as a blurred, patchy red background due to the reflection of red light

Trial images were likewise obtained with 365nm excitation and both with and without a 452nm bandpass emission filter (compare Fig. 5D, F with Fig. 5C, E). Fluorescent roots were clearly visible (Fig. 5C, D). There was little background fluorescence or reflectance, irrespective of the presence of the emission filter. However, the emission filter lowered the contrast by reducing the brightness of the fluorescing root. A more complex arrangement of exposed roots that are both weakly and strongly fluorescent can be seen in Figure 5E, F. There was more background in these images, possibly because the fluorescent roots are of sufficient abundance to illuminate the soil. However, there was a high contrast between the roots and the background, a contrast that was reduced by bandpass filtering. For this reason, bandpass emission filtering was not used for subsequent imaging.

### Calibration of image analysis software: correlation of BlueBox output with root length density

**Fig. 6.**
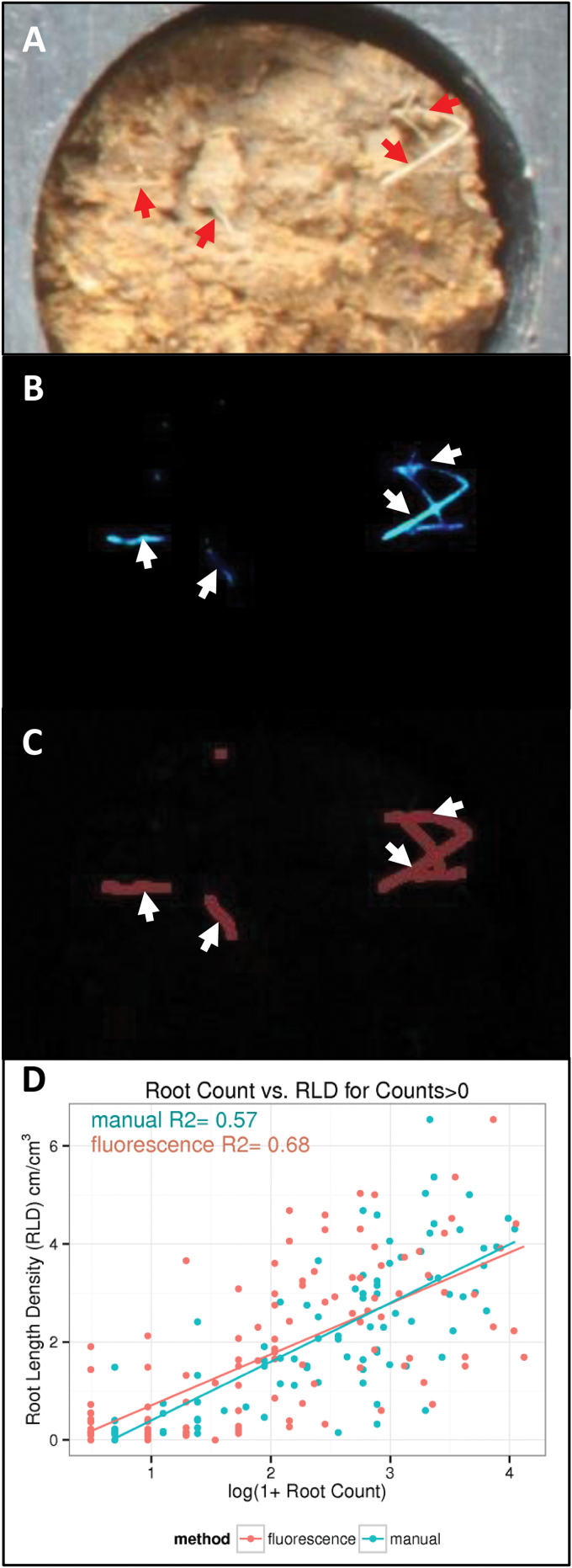
**Visible and automated identification of roots.** (A) A visible image of a soil core segment in the BlueBox cassette. Visible roots are highlighted with red arrows. (B) A fluorescence image of the same root segment (contrast thresholds adjusted). The same roots are highlighted with white arrows. (C) Roots identified within the fluorescence image by the image analysis algorithm. The position of the roots is highlighted with white arrows. (D) Correlation between root counts and root length density as collected manually by a human operator from visible images (in blue) and by the fluorescence imaging system processed with image analysis software (in red). Both datasets were derived from images of the same soil cores in the imaging cassettes.

The correlation between the core-break counts in the field and the true root length in the core fragments was calculated. Figure 6 illustrates the differences between the manual core-break and BlueBox approaches. Figure 6A shows an exposed core face in the BlueBox cassette. Were this face being examined manually in the field, the operator would record four separate roots (highlighted with red arrows in the figure). When imaged with the BlueBox, those four roots were apparent (following thresholding of the image; Fig. 6B). When processed by the image analysis software, those four roots were detected (along with an additional object at the top of the image; Fig. 6C). The software outputs a count of the number of fluorescent objects in the image and the combined length of those objects in the image. Hence, in the processed image the two roots on the right are counted as a single overlapping object. The image analysis software was unable to distinguish two overlapping roots when counting, where a human operator may be able to do so. Furthermore, the object at the top centre of the image may be a false-positive detection, despite the application of filters designed to remove spurious objects.

In the parameterization process, the sensitivity of the detection of fluorescent objects and the sensitivity of the filtering processes to remove false positives were varied, and the consequences for the correlation were determined. Hence, the parameters were determined empirically and found to produce a reasonable approximation of what could be visibly detected. The correlation achieved by the BlueBox was 0.68 (Fig. 6).

Every fluorescence image was accompanied by a visible image, and the roots apparent in these images were counted manually by a human operator, analogously to how the roots would be counted were the soil cores being manually processed in the field (albeit without the time pressures or discomforts of field work). These counts were likewise correlated against the washed root length density data, but the correlation achieved was 0.57, inferior to that of the BlueBox.

### Impact of the BlueBox on processing speed

The speed of soil core processing in the field was recorded across experiments. A team consisting of five operators manually processing samples can process 130 cores/day, or 3.71 cores/hour/person (Table 1). With the BlueBox, a four-person team could process 120 cores/day, or with three people 100 cores/day, or 4.29 and 4.76 cores/hour/person, respectively (Table 1).

**Table 1.** **Productivity impacts of the BlueBox system and estimated impacts on sampling costs.** Cores are 2 m soil cores broken into 40 segments (of 5cm) and imaged on both faces (i.e. 80 faces per core). Calculations are based upon 7 hours/working day; a typical technician cost at the location where the project was conducted of US$34.22/hour; project phenotyping 400 genotypes, four replicates and four cores/replicate at two sites over 3 years; cost of push press US$40000; cost of BlueBox US$5000. Estimates do not include the cost of moving equipment between sites and assume use of a local workforce, as accommodation costs for a mobile workforce would be prohibitive. Site harvest periods would have to be non-concurrent as harvest duration would be 10–11 weeks.

Team size	Cores/day	Cores/hour/person	Cost/core (% of five people team value)
5 people	130	3.71	100%
4 people + BlueBox	120	4.29	86%
3 people + BlueBox	100	4.76	78%
**Estimated sampling costs** (% of five people team value)	**Fixed**	**Operating**	**Total**
5 people	100%	100%	100%
4 people + BlueBox	113%	86%	88%

## Discussion

This study developed a portable and automated imaging system to capture root traits in soil cores and reduce the labour costs associated with direct root sampling. Utilizing the autofluorescence of roots was investigated as one approach to generate high-contrast images that could be processed with image analysis algorithms. Root autofluorescence was found to provide a strong contrast between roots and soil, and high-contrast images of roots in soil were obtained by excitation of the core-break face with UV light. An image analysis algorithm was created that was capable of identifying roots on the core faces. The algorithm-derived root counts correlated with washed root length densities with a correlation coefficient of 0.68; when manual root counting was performed the correlation was inferior at 0.57. In a four-person team the BlueBox improved the labour efficiency of the procedure by 16%.

### Contrast between BlueBox and the manual core-break approach

While the use of the BlueBox reduced the daily throughput of cores, the reduced labour required raised the productivity of the operations. The effect of the BlueBox on sampling costs for root phenotyping is estimated in Table 1. The calculation assumes the phenotyping of 400 genotypes, four replicates at four cores per replicate at two sites over 3 years. The equipment costs are conservatively estimated at US$40000 for the push press and US$5000 for the BlueBox, but assume standard research station infrastructure, such as compatible tractors. For a project of that scale, the sampling costs with the BlueBox would be 88% of the costs of a five-person team using a standard approach. Hence, the BlueBox investment represents 1% of the project budget but delivers a 12% reduction in costs.

The correlation with the washed root length density indicates that the BlueBox approach is as effective as the manual core-break approach. However, the root counts derived by the image processing software differ from the manual counts (Fig. 6), and both are likely to differ from the true number of intersections. Furthermore, as with the manual core-break technique, the efficacy of the BlueBox is expected to vary between sites with different soil conditions (Wasson *et al.*, 2014) and will vary with species. This is because the core-break method assumes that roots are randomly distributed in three dimensions (see Introduction; van Noordwijk *et al.*, 2000), but in a structured soil, pores, cracks, and other soil features will influence how the plants grow (Wasson *et al.*, 2014; White and Kirkegaard, 2010). As this is fundamental to the relationship between the core-break count and the root length density, it will affect the BlueBox as much as manual methods of counting. The calibration process undertaken in this study, whereby a subset of cores is retained for root length density determination, should be employed each time a new species and site is used.

The prospect that soils high in organic matter may create a fluorescent background, rendering the BlueBox ineffective, seems remote. Fluorescence spectroscopy is used in the study of soluble organic matter in soil (Senesi *et al.*, 1991) and aquatic contexts (Coble *et al.*, 2014), but the fluorescence of soil itself (rather than prepared extracts) has been described as ‘meagre’ (Gallagher, 1949).

The BlueBox may improve the comparison of studies between sites in important ways. First, in lighter soils there is less visible contrast between roots and soil, making it harder for operators to make an accurate count. This problem does not affect the fluorescence contrast and hence the BlueBox. Secondly, and perhaps unsurprisingly, operators are inconsistent in their counts, particularly when working under arduous field conditions and for extended periods of time. The BlueBox offers a greater degree of standardization, with known parameters for how images are collected and processed, and a permanent record (the images), which can be reprocessed consistently if required. Finally, although this point may seem trivial, there was a (unfortunately unquantifiable) boost in morale among operators who were using the BlueBox rather than manually counting roots. This is important for phenomics experiments, where sampling campaigns may take weeks, and may even encourage more investigators to undertake root phenotyping studies in the field.

Sources of error and delay were revealed in the operation of the imaging system. As noted in the Materials and methods, a time variable setting with manual focusing was used. This meant the aperture was being set automatically by the camera, which was an unwanted source of variation between the images. Future experiments should use a full manual setting with f22 and f10 (f-stop, a measure of relative aperture) for visible and fluorescence images, respectively. A white lighting system should also be installed to provide consistent illumination for the visible images.

Furthermore, processing the cores into the cassettes required human operators and resulted in uneven fragment sizes and break surfaces. This may have reduced the correlation between the machine-generated counts and the root length density in the fragment. It also increased the amount of effort required to flip the imaging cassette to image both ends of the core fragment. Automation of the cassette loading process would further reduce the labour requirements and may improve the throughput of the system.

Root architecture is highly influenced by edaphic factors. This is likely to drive the plot and core variation, and the high levels of residual variation in the mixed model. Measurements of these edaphic factors, such as soil texture, water, and nutrient status, within each core could be used as a covariate in the modelling of the root measurements. Given the effort required to acquire the soil cores, processing them for root information alone seems a missed opportunity. Efforts should be made to simultaneously measure edaphic factors and root traits.

### Use of fluorescence

Recently, a laser-induced fluorescence imaging system has been used to detect autofluorescence in maize and okra roots growing in rhizoboxes and on filter paper (Alony and Linker, 2013). Using the high-intensity illumination of the pulsed UV laser and coupling it with an intensified gated camera (which captured only the fluorescence generated after the UV pulse), the authors were able to detect the autofluorescence even under ambient light. The nature of the autofluorescence differed in its rate of decay—which could also be measured by the system—and was used to differentiate between okra and maize roots. This suggests that fluorescence techniques could be used for taxa identification in mixed stands or to distinguish weed from crop roots in an agricultural context (Rewald and Meinen, 2013; Rewald *et al.*, 2012).

The intensity of root autofluorescence has been shown to vary along the length of the root, and is typically brightest at the tip (Alony and Linker, 2013; Dyer and Brown, 1983). This zone of intensity has been observed moving up the root towards the seed as a seedling root was left to dry on a paper surface (Alony and Linker, 2013). Zones of intense autofluorescence have been observed where new roots are emerging (Alony and Linker, 2013) and where nodules are emerging in legume roots (Mathesius *et al.*, 1998). This variation may reduce the accuracy of root quantification with the BlueBox, but it could also be developed as a means by which the age of the roots is estimated along with their depth and distribution. Likewise, previous attempts to estimate the live/dead status of roots from their fluorescence intensity have been challenging (Smit and Zuin, 1996; Wang *et al.*, 1995), but it may be possible to achieve better results with fluorescence spectrometry.

The majority of plant root systems studied have exhibited fluorescent properties (Goodwin and Kavanagh, 1948), so this technique should be broadly applicable. However, variation of fluorescence has been observed within a species; for example, an evaluation of 572 soybean genotypes identified 59 without root fluorescence (Delannay and Palmer, 1982). The possibility of variation of fluorescence within a breeding population should be considered when the BlueBox is employed.

### Prospects for development

The BlueBox utilizes simple root fluorescence to produce a measurement that can be correlated with root length density, but the accuracy of the technique may be reduced by variation in fluorescence governed by root age, health, infection, and physiology. However, it may be possible to adapt the system to take spectroscopic measurements of the fluorescing roots to capture this variation for biological insights. Rewald and Meinen (2013) reviewed spectroscopic techniques for analysing root biomass, species, and vitality. Mid-infrared and near-infrared techniques have been used to distinguish roots in mixed stands, and to distinguish live and dead roots. These techniques can be disrupted by high water contents; however, Fourier-transform Raman spectroscopy offers another spectroscopic alternative that is less influenced by water content. The majority of these studies have been based on rhizobox and minirhizotron studies, but there is scope for adapting them for the analysis of soil cores in the field.

Using these techniques, it may be possible to gather information not only on the roots and their status but also on their interaction with the rhizosphere. A laser-induced fluorescence detection system was able to detect autofluorescent compounds exuded into the ‘rhizosphere’ of the roots (in that case, in perlite), to the extent that it was able to detect fluorescently the rhizosphere of a maize root that was otherwise obscured in the visible image by a layer of perlite (Alony and Linker, 2013). The authors speculate that the identity of the autofluorescent compounds could be determined by their decay time profiles (Alony and Linker, 2013). Likewise, the system could be used to detect compounds interacting with fluorescent dyes sprayed on to the interior of the rhizobox.

### Conclusion

The results of this study demonstrate that fluorescence imaging and image analysis algorithms can be used to derive root measurements from soil cores, in a process analogous to soil coring with core-break counting. The cost of the instrument would ordinarily deliver a return on investment in reduced labour costs in a root-phenotyping exercise. Future studies might explore whether the platform can be used to capture more information about root health and other root traits in the field.
